# Complete genome sequence of *Bacillus subtilis* A1, a *Malus micromalus* endophytic strain inducing pigment production of phylogenetically diverse panel fungi

**DOI:** 10.1128/mra.01037-24

**Published:** 2025-03-20

**Authors:** Zurong Shi, Qian Yang, Hong Rong, Qingwei Wang

**Affiliations:** 1 School of Biological Engineering, Huainan Normal University 165081 , Huainan, Anhui, China; 2 School of Medicine, Anhui University of Science and Technology 91594 , Huainan, Anhui, China; University of California Riverside, Riverside, California, USA

**Keywords:** *Bacillus subtilis*, biocontrol, pigment production

## Abstract

Endophytic bacterium *Bacillus subtilis* A1, isolated from the leaves of *Malus micromalus*, exhibits antagonistic abilities against several pathogenic fungi and meanwhile induces production of pigments in these phylogenetically diverse panel fungi. The complete genome of A1 was sequenced and annotated, which is 4,088,362 bp in length with 46.32% GC content.

## ANNOUNCEMENT

**Fig 1:**
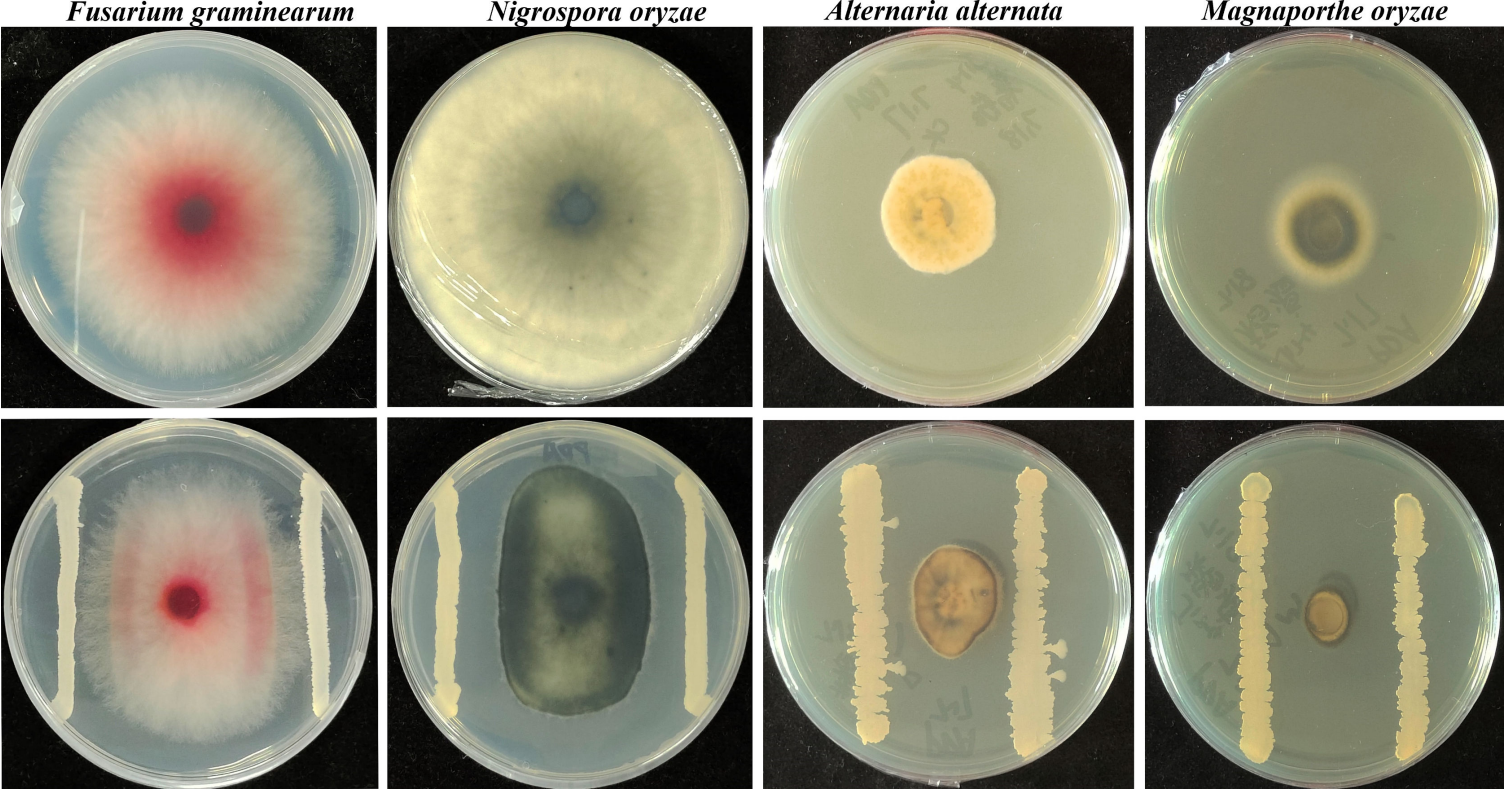
When grown in coculture on agar plates, *Bacillus subtilis* A1 inhibits growth of four pathogenic fungi and induces the production of pigments in these phylogenetically diverse panel fungi.

Fengycin is one of the three dominant cyclic lipopeptides in *Bacillus* spp., acting as weapons against a range of yeasts and filamentous fungi at higher concentrations or as communication signals at sub-inhibitory concentrations, inducing changed phenotypes of fungi (1). To screen endophytic bacteria, we collected healthy *Malus micromalus* (32°36′42.1″N and 116°57′54.0″E) leaves in Huainan, Anhui, China. These leaves were surface sterilized with 75% ethanol and sodium hypochlorite solution (2% available Cl) for 3 and 5 min, respectively. Then, these leaves were placed in mortar to grind into pieces and homogenated with 2 mL ddH_2_O, and 100 µL of the suspension was planted on LB agar plates for bacteria isolation. Strain A1 was obtained owing to its great antagonistic effect and its production inducing localized pigment production accumulation of several fungal pathogens via confront culture (Fig. 1), and this strain was further identified as *Bacillus subtilis* via taxonomic analysis (unpublished data). To gain a comprehensive understanding of the biocontrol potential and the underlying mechanism of the shifts in secondary metabolite production of many phylogenetically diverse panel fungi during interaction with strain A1, we described its complete genome sequence.

A single colony of strain A1 was inoculated in 10 mL of the LB broth at 28°C with 200 rpm agitation for 24 h. Genomic DNA was extracted from the bacterial culture using the HiPure Bacterial DNA Kits (Magen, China) and assessed its quality by using Qubit (Thermo Fisher Scientific, Waltham, MA) and Nanodrop (Thermo Fisher Scientific, Waltham, MA). Qualified DNA was used for whole genome sequencing through the PacBio Sequel and Illumina NovaSeq 6000. PacBio sequencing was performed on platforms of the Pacific Biosciences Sequel (PacBio, Menlo Park, CA) and Oxford Nanopore PromethION (Oxford Nanopore Technologies, Oxford, UK), which generated 110,195 subreads with an average read length of 1,3278.8 bp (N50, 14,509 bp) were selected for assembly *de novo* by using the diploid-aware long-read assembler FALCON-Unzip of FALCON (version 0.3.0) (2) and Flye (version 2.8.1-b1676) (3), respectively. Alternatively, a short-read library was prepared using the NEBNext Ultra DNA Library Preparation Kit (NEB, USA) and sequenced using an Illumina NovaSeq 6000 platform with 150 bp paired-end reads. Size selection was performed using AMPure beads, removing fragments smaller than 1,000 bp, generating a total of 10,291,714 reads and 154.3 Mbp of sequencing data (Q30(%) = 92.52). The Illumina sequencing data were subjected to correct the genome sequences of PacBio sequencing to improve the quality of its assembly, and then the final genome sequences were determined via Pilon (version 1.23) (4). All tools were applied with default parameters unless noted otherwise. The open reading frames (ORFs) were predicted using the NCBI prokaryotic genome annotation pipeline (5). Noncoding RNAs, such as rRNAs prediction, was carried out using rRNAmmer (version 1.2) (6), while tRNAs and sRNAs were identified through using tRNAscan (version 1.3.1) (7) and cmscan (version 1.1.2) (8), respectively. Gene clusters of secondary metabolites were predicted using antiSMASH (version 4.1.0) (9).

The complete genome of A1 is composed of a circular chromosome of 4,088,362 bp with 46.32% G + C content and 3,825 coding sequences. The additional features of this genome were summarized in Table 1. Thirteen gene clusters were predicted associated with secondary metabolite production, including one NRPS (nonribosomal peptides), two terpenes, one T3pks, two Transatpks, two Transatpks-Nrps, one Bacteriocin-nrps, one otherks, one phosphonate, and one other.

**TABLE 1:** Genomic information for *Bacillus subtilis* A1

Bacterial strain	*B. subtilis* A1
Total length (bp)	4,088,362
% GC content	46.32%
Genes	3,954
Proteins	3,811
rRNA	28
tRNA	87
sRNA	14
Pseudogenes	14
CRISPR arrays	2
Prophages	4

## Data Availability

The genomic data of *B. subtilis* A1 were deposited in the NCBI under the BioProject accession number PRJNA1051337, the BioSample accession number SAMN38770643, the SRA accession number SRR31560146, and the GenBank accession number CP141181.
